# Mitochondrial Genome Evolution in a Single Protoploid Yeast Species

**DOI:** 10.1534/g3.112.003152

**Published:** 2012-09-01

**Authors:** Paul P. Jung, Anne Friedrich, Cyrielle Reisser, Jing Hou, Joseph Schacherer

**Affiliations:** Department of Genetics, Genomics and Microbiology, University of Strasbourg/Centre National de la Recherche Scientifique, Unité Mixte de Recherche 7156, Strasbourg, France

**Keywords:** mitochondrial DNA, intraspecific diversity, population structure, purifying selection, *Lanchancea kluyveri*

## Abstract

Mitochondria are organelles, which play a key role in some essential functions, including respiration, metabolite biosynthesis, ion homeostasis, and apoptosis. The vast numbers of mitochondrial DNA (mtDNA) sequences of various yeast species, which have recently been published, have also helped to elucidate the structural diversity of these genomes. Although a large corpus of data are now available on the diversity of yeast species, little is known so far about the mtDNA diversity in single yeast species. To study the genetic variations occurring in the mtDNA of wild yeast isolates, we performed a genome-wide polymorphism survey on the mtDNA of 18 *Lachancea kluyveri* (formerly *Saccharomyces kluyveri*) strains. We determined the complete mt genome sequences of strains isolated from various geographical locations (in North America, Asia, and Europe) and ecological niches (*Drosophila*, tree exudates, soil). The mt genome of the NCYC 543 reference strain is 51,525 bp long. It contains the same core of genes as *Lachancea thermotolerans*, the nearest relative to *L. kluyveri*. To explore the mt genome variations in a single yeast species, we compared the mtDNAs of the 18 isolates. The phylogeny and population structure of *L. kluyveri* provide clear-cut evidence for the existence of well-defined geographically isolated lineages. Although these genomes are completely syntenic, their size and the intron content were found to vary among the isolates studied. These genomes are highly polymorphic, showing an average diversity of 28.5 SNPs/kb and 6.6 indels/kb. Analysis of the SNP and indel patterns showed the existence of a particularly high overall level of polymorphism in the intergenic regions. The dN/dS ratios obtained are consistent with purifying selection in all these genes, with the noteworthy exception of the *VAR1* gene, which gave a very high ratio. These data suggest that the intergenic regions have evolved very fast in yeast mitochondrial genomes.

The mitochondrion is a central organelle, which participates in key cellular functions such as respiration, metabolite biosynthesis, ion homeostasis, and apoptosis. These essential cellular processes are conserved in all eukaryotic organisms, from humans to yeasts. However, the mitochondrial DNA (mtDNA) has been found to show a greater rate of mutation than the nuclear DNA. The mtDNA mutation rate and spectrum have been determined by performing mutation-accumulation experiments on a wide range of model organisms, including *Caenorhabidtis elegans* (Denver *et al.* 2000), *Caenorhabidtis briggsae* (Howe *et al.* 2010), *Pristionchus pacificus* (Molnar *et al.* 2011), *Drosophila melanogaster* (Haag-Liautard *et al.* 2008), and *Saccharomyces cerevisiae* (Lynch *et al.* 2008). The mutation rates are high (around of 10^−8^ to 10^−7^ per site per generation) in all these species, but they vary from one species to another.

Studies on yeasts, especially those belonging to the Hemiascomycetes phylum, have contributed considerably to our understanding of interspecific mitochondrial (mt) genome evolution. Complete mitochondrial genome sequences are now available on more than 40 hemiascomycetous species covering a broad evolutionary range (Solieri 2010; Gaillardin *et al.* 2011). The availability of these genome sequences provided an opportunity for us to investigate the changes in the patterns of mtDNA organization and genome architecture from the evolutionary perspective. The size of mt genomes varies between yeast species, ranging, for example, from 11 to 85 kb in *Hanseniaspora uvarum* and *S. cerevisiae*, respectively (Foury *et al.* 1998; Pramateftaki *et al.* 2006). Despite this high level of diversity, a core set of genes is conserved. This core contains the genes encoding the apocytochrome b (*COB*), three subunits of ATP synthetase (*ATP6*, *ATP8*, and *ATP9*) and three subunits of cytochrome oxidase (*COX1*, *COX2*, and *COX3*). In addition, mtDNAs encode both large (*LSU*) and small (*SSU*) rRNA genes and a complete set of tRNA genes. However, some variability of the gene content is also observed among yeasts. Several genes, including those encoding *VAR1* (a ribosomal protein), *RPM1* (the RNA subunit of the mitochondrial RNaseP), and the seven NADH:ubiquinone oxidoreductase (complex I) subunits do not occur consistently in all yeast mt genomes. The fact that the gene order varies significantly indicates that a large number of rearrangements has occurred with time (Bouchier *et al.* 2009; Jung *et al.* 2009, 2010; Valach *et al.* 2011). A few exceptions have been observed, however, where the synteny is conserved across species. The gene order is completely conserved between *Candida parapsilosis*, *Candida orthopsilosis*, and *Candida metapsilosis*, which are closely related species as well as between species of the *Yarrowia* clade, which are phylogenetically more distantly related species (Kosa *et al.* 2006; Gaillardin *et al.* 2011).

The many complete yeast mt genome sequences published by now have shown the existence of considerable variations and a wide range of genome architectures among species. Despite the existence of this ever-increasing body of data, relatively little is known so far about the mitochondrial genome variations existing within single yeast species. Due to the lack of intraspecific mitochondrial data, the evolution of mtDNA has not yet been completely elucidated. Because the differences between the mt genomes of various wild yeast isolates have not so far been documented, it was proposed here to investigate the patterns of mtDNA diversity present in a single yeast species by conducting a comprehensive survey on a set of *Lachancea kluyveri* (formerly known as *Saccharomyces kluyveri*) yeast isolates. *L. kluyveri* strains can be isolated from various sources such as soil, *Drosophila*, and tree exudates. Unlike *S. cerevisiae*, this species has not been domesticated (Liti *et al.* 2009; Schacherer *et al.* 2009). In addition, *L. kluyveri* is a protoploid *Saccharomycetaceae*, which means that it diverged from the *S. cerevisiae* lineage before undergoing ancestral whole-genome duplication (WGD) and is therefore a pre-WGD yeast species (Souciet *et al.* 2009). Interestingly, in species belonging to the *Saccharomyces* genus that underwent WGD, the nuclear genes acting in the mitochondria show a greater rate of evolution than the other genes, which suggests that the mitochondrial functions have weakened in these post-WGD yeasts (Jiang *et al.* 2008). These findings can be correlated with the fact that most post-WGD species can live without a functional mt genome with their ability to generate petite mutants (Merico *et al.* 2007). Unlike the *Saccharomyces* species, *L. kluyveri* is petite-negative and mainly ferments sugar in the absence of oxygen (Piskur *et al.* 1998; Møller *et al.* 2001). We therefore expected *L. kluyveri* to be a suitable model organism for studying intraspecific yeast mt genome evolution.

Here, we present the complete mt genome sequences of 18 *L. kluyveri* strains, which were isolated from various geographical locations (in North America, Asia, and Europe) and ecological niches (*Drosophila*, tree exudates, and soil). We first assembled the mt genome of the NCYC 543 reference strain (also known as CBS 3082), the nuclear genome of which was recently sequenced (Souciet *et al.* 2009). This genome is 51,525 bp long and contains the same set of 35 genes as those described in *Lachancea thermotolerans* (CBS 6340 strain), its closest relative (Talla *et al.* 2005). To provide a better picture of the genetic diversity among *L. kluyveri* species, we compared the mtDNA of these 18 isolates. This species is composed of two geographically defined lineages corresponding to the North American and European isolates. The genomes are syntenic, but the size of the mtDNA and the intron content differ. Whole-mtDNA genome analysis clearly showed the presence of a greater rate of SNPs and indels in the intergenic regions than in the coding regions. The dN/dS ratios obtained clearly suggested that purifying selection purged most nonsynonymous differences from the protein-coding genes. Only the dN/dS ratio obtained in the case of the *VAR1* gene was exceptionally high, possibly due to changes in the functional constraints to which this gene was exposed.

## Material and Methods

### Strains and DNA preparation

Strains to be sequenced were selected so as to maximize the range of sources and the types of location from which they were isolated. The list of the 18 *L. kluyveri* strains used in this study, which were purchased from various yeast culture collections, is given in supporting information, Table S1.

Yeast cell cultures were grown overnight at 30° in 20 mL of YPD medium up to the early stationary phase. Total genomic DNAs were subsequently extracted using the QIAGEN Genomic-tip 100/G in line with the manufacturer’s instructions.

### Sequencing and assembly

Genomic paired-end Illumina sequencing libraries were prepared with a mean insert size of 280 nt. The 18 libraries were multiplexed in 2 Illumina HiSeq2000 lanes for sequencing. Paired-end reads from 102 nt, six of which were dedicated to the multiplex tag, were obtained, amounting to 149,308,900 paired-end reads in all. FASTX-Toolkit (http://hannonlab.cshl.edu/fastx_toolkit/) was first used with options “-t 20 -l 50” to clean the reads. Two independent *de novo* assemblies (ace63 and ace75) were then constructed with each strain using SOAPdenovo, version 1.05 (Li *et al.* 2010), with two different Kmer sizes (soapdenovo63mer with option –K 63 and soapdenovo127mer with –K 75).

### Mitochondrial reference sequence construction and annotation

The assemblies obtained with strain NCYC 543 were used to construct a mitochondrial reference sequence corresponding to the *L. kluyveri* species. We identified mitochondrial contigs and scaffolds within ace63 and ace75 assemblies by performing similarity searches with the BLAST suite of programs (Altschul *et al.* 1997) using *L. thermotolerans* mitochondrial gene sequences as the query (Talla *et al.* 2005). A total number of 135 scaffolds and contigs were highlighted in these two independent assemblies, among which 98 were redundant. The 37 remaining sequences were compared with MUMmer 3.0 (Kurtz *et al.* 2004) to detect overlapping segments and the alignment of all these sequences was refined manually to obtain a single contig.

The protein-coding genes as well as the large and small subunits of the ribosomal RNA genes were exactly located on the complete mitochondrial sequence by performing BLASTN searches with *L. thermotolerans* genes. The *COX1* and *COB* intronic regions were refined manually based on the conservation of the protein sequences. The position of introns within the *LSU* genes was determined based on the boundaries defined for both *S. cerevisiae* and *L. thermotolerans* species (Jacquier and Dujon 1983).

The same procedure was used to assemble and annotate the complete mitochondrial sequences of the 55-86.1, 77-1003, CBS 6547, and CBS 5828 strains for intraspecific comparison purposes. We obtained complete mitochondrial contigs for the 55-86.1 and 77-1003 strains. By contrast, there are one gap of 49 bp and two gaps of 30 and 40 bp in the scaffold of CBS 5828 and CBS 6547, respectively.

Sequences are available under EMBL accession numbers HE664110 for the NCYC 543 strain, HE774680 for the 55-86.1 strain, HE664111 for the 77-1003 strain, HE774664 for the CBS 6547 strain, and HE664112 for the CBS 5828 strain.

### Phylogenetic studies

The coding sequences of *ATP6*, *ATP8*, *ATP9*, *COB*, *COX1*, *COX2*, *COX3*, and *VAR1* genes were extracted from the initial ace63 assembly of the *L. kluyveri* strains by performing similarity searches with NCYC 543 gene sequences as the query. The sequences of all the genes were recovered in all strains except for the CBS 10368 strain, in which *VAR1* could not be completed. The *VAR1* gene was therefore removed from the phylogenetic analysis. The nucleotide sequences of these genes were automatically aligned with MUSCLE (Edgar 2004) and manually inspected before being concatenated.

Based on these 5475 aligned positions, phylogenetic relationships among *L. kluyveri* strains were analyzed using neighbor-joining (using the Kimura two-parameter substitution model), maximum likelihood (using the Hasegawa-Kishino-Yano 85 substitution model), and Bayesian (10,000 generations, sampling trees every 10 generations, discarding the first 250 trees as a burn-in) methods. These analyses were performed with SeaView (Gouy *et al.* 2010), PhyML (Guindon and Gascuel 2003), and MrBayes (Dereeper *et al.* 2008), respectively. Bootstrap analyses (1000 replications) were used to assess the confidence level of each node for the Neighbor-joining method.

### Selection and dN/dS ratios

The dN/dS ratios were calculated using CODEML model in PAML package version 4.4b (Yang 2007). We used a tree-based maximum-likelihood method. To summarize, coding sequence multi-alignments was generated for each of the eight protein-coding genes among the set of 18 *L. kluyveri*, as described previously. Based on the alignments, neighbor-joining trees were constructed using ClustalX and then labeled manually considering the branch-length and genetic proximity of the strains. Estimates of the dN/dS ratios were then calculated for each gene.

### Population structure

The estimation of the number of population clusters was performed with the *Structure* program, version 2.3.1 (Pritchard *et al.* 2000), based on the 180 polymorphic positions detected in the seven protein-coding sequences previously used for the phylogenetic analyses. We ran the *Structure* program using the admixture model with the population number parameter *K* set from two to four, on 100,000 replicates after a burn-in of 100,000 iterations and did not incorporate any prior population information in these analyses.

## Results

To investigate the intraspecific mtDNA variations present in yeast, we sequenced complete mitochondrial genomes from a set of 18 *L. kluyveri* isolates. These strains were isolated in various countries worldwide from ecological sources of various kinds (insect guts, tree exudates, and soil; Table S1). The complete mt genome sequences were determined directly using high-throughput sequencing methods. Illumina libraries were constructed from genomic DNA isolated from the various strains and run on two lanes of a flow cell on an Illumina HiSeq 2000 using 102-bp paired-end sequencing methods. In this way, a coverage of 100% was obtained and the average read depth was ∼1145x.

### Mitochondrial genome of *L. kluyveri*

**Figure 1:**
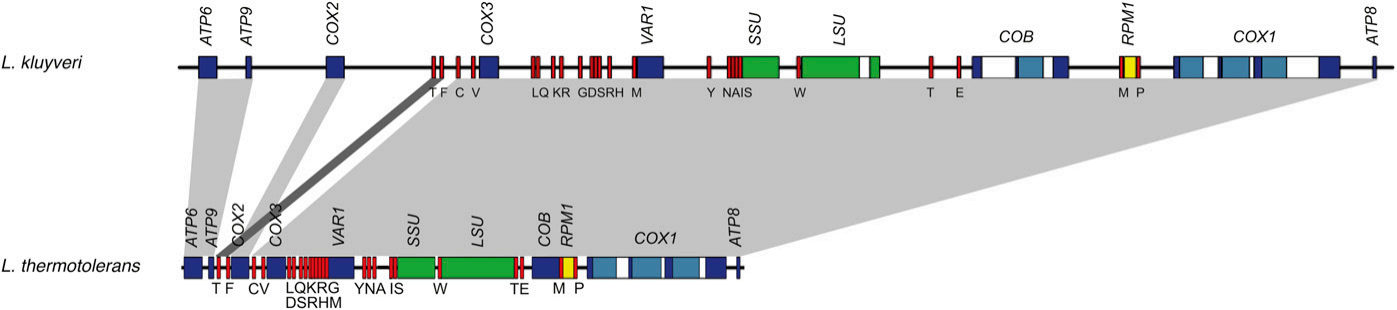
Overview of the organization of the mtDNA of *L. kluyveri*. The mtDNA of *L. thermotolerans* is also shown so as to be able to compare the structure and the synteny. Protein-coding genes and tRNA and rRNA genes are presented in dark blue, red, and green, respectively. Coding introns are represented in light blue and noncoding introns in white. The *RPM1* gene is presented in yellow.

The nuclear genome of the reference NCYC 543 strain (also known as CBS 3082) was previously sequenced, but the mt genome was not sequenced (Souciet *et al.* 2009). This strain was isolated from the intestinal canal of *Drosophila pinicola* in the Yosemite National Park of California in the United States. We first assembled the mt genome. When we used SOAPdenovo, assembly of the reads yielded a single scaffold, which is 51,525 bp long and shows a GC content of about 15% (Table S2). This mitochondrial sequence is more than twice the size of that obtained in the closely related species *L. thermotolerans* [CBS 6340 strain (Talla *et al.* 2005)]. Despite this difference in size, the same set of 35 genes is encoded by the two genomes, both being transcribed from only one DNA strand (Figure 1). Among these 35 genes, eight are protein-coding genes and 27 are noncoding RNA genes. The protein-coding genes encode three subunits of the cytochrome c oxidase (*COX1*, *COX2*, and *COX3*), three subunits of the ATP synthase (*ATP6*, *ATP8*, and *ATP9*), the apocytochrome b (*COB*) and a ribosomal protein (*VAR1*). The noncoding RNA genes comprise two genes encoding the small and large RNA subunits of the ribosome (*SSU* and *LSU*, respectively), the *RPM1* gene that codes for the RNA subunit of the RNaseP and 24 tRNA genes. The *RPM1* gene was detected using three conserved regions among ascomycete fungi, named CRI, CRIV, and CRV (Seif *et al.* 2003). This gene is located between tRNA^Pro^ and tRNA^Met^: this location is the same as that previously found to occur in *S. cerevisiae* and *L. thermotolerans* (Foury *et al.* 1998; Talla *et al.* 2005). The set of tRNA genes includes at least one tRNA for each of the 20 amino acids and sufficed to be able to decipher the fungal mitochondrial genetic code. This genetic code differs from standard code because mtDNA encodes a tRNA^Trp^ able to decode UGA codon as it has been reported for several yeast species including *L. thermotolerans* (Talla *et al.* 2005).

Complete analysis of gene conservation order between the mt genomes of *L. kluyveri* and *L. thermotolerans* showed that these two sequences are almost completely syntenic (Figure 1). With the exception of the two tRNAs surrounding the *COX2* gene, these two genomes are colinear, which suggests that a very low rate of gross chromosomal rearrangements of mtDNA has occurred in the *Lachancea* clade (Figure 1). Despite the high levels of synteny observed, comparisons between entire mt genomes of *L. kluyveri* and *L. thermotolerans* showed the existence of considerable genomic variability, especially in the noncoding sequences. This variability explains the size differences observed, for example. The twofold size difference is found to be correlated with the relative size of the intergenic regions, which ranges from 22.3% in *L. thermotolerans* to 41% in *L. kluyveri* (Table S2), as well as with the difference between the intron content of three genes: *COB*, *COX1*, and *LSU*. Unlike the *L. thermotolerans COB* gene, which is intronless, that of *L. kluyveri* harbors two introns. In addition, although the *COX1* genes of those two species both contain three introns, the third one is almost two times larger in *L. kluyveri* than in *L. thermotolerans* (Figure 1). All the introns present in the protein-coding genes of *L. kluyveri*, apart from the first intron in the *COB* gene, encode endonucleases belonging to the LAGLIDADG family of group I introns (Haugen *et al.* 2005). An intron was also detected in the *LSU* gene, with the same downstream boundary as that described in *S. cerevisiae* and an upstream boundary containing only one additional nucleotide (Jacquier and Dujon 1983).

### Intraspecific diversity in the mitochondrial coding regions

To explore the intraspecific variability, DNA sequence diversity in the coding region was first compared among 18 *L. kluyveri* isolates. A total number of 208 polymorphic positions (3.16% of the sites on the 6591 bp) showed a nucleotide substitution. The frequency of polymorphism is 0.0098 per bp on average. Among the 1101 SNPs identified in coding genes, 171 are nonsynonymous and 930 are synonymous (Table S3). This imbalance strongly suggests that purifying selection has purged the nonsynonymous differences from the protein-coding genes.

**Figure 2:**
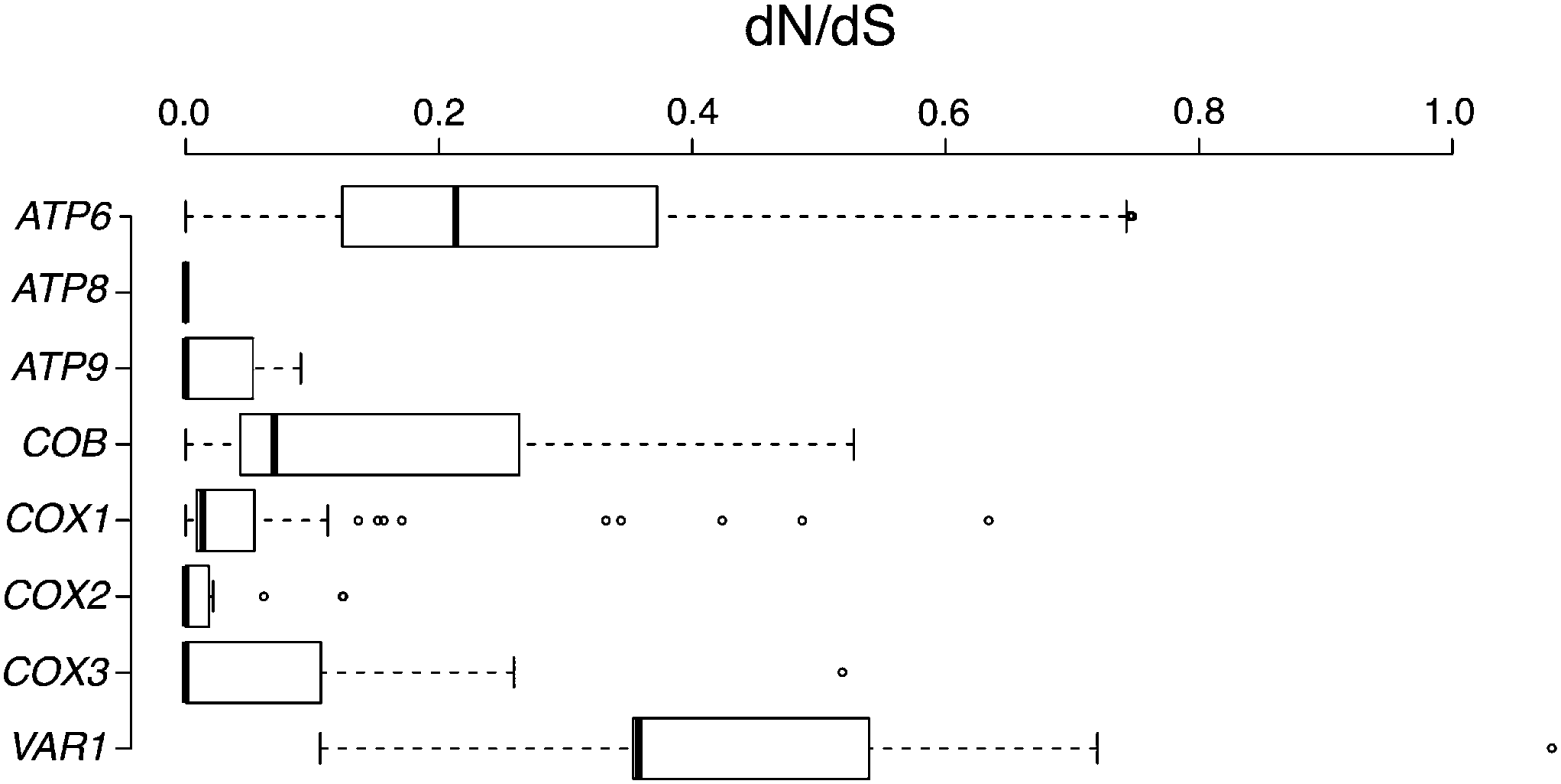
Box-plot comparisons of the dN/dS ratio [ω] estimated in the various mt genes and based on pairwise alignments.

To determine the effects of purifying selection, we then quantified the selective constraints present in the mt genomes by estimating the ratio of nonsynonymous (dN) to synonymous (dS) substitution rates ω = dN/dS. We calculated the average ω ratio in each of the coding genes (Figure 2). In all the cases studied, dN was lower than dS, signature of strong purifying selection of mitochondrial genes (Figure S1). Median values of ω lower than 1 were obtained in all the genes, but the values differed from one gene to another (Figure 2). The *ATP8*, *ATP9*, and *COX3* gene ratios were lower than average. In addition, the *ATP6* and *VAR1* genes were found to be characterized by a dN/dS ratio, which is well above average, giving a median value of 0.21 and 0.35, respectively. This finding might potentially be attributable either to positive selection or to a reduced constraint level on these genes. The results observed for *VAR1* gene is interesting not only because of the elevated dN/dS ratio but also because we observed an exceptionally low dS value (the lowest in this set) and high dN value than average (the second highest in this set) (Figure S1).

### Intraspecific diversity of the intron content

The mitochondrial intron content is known to vary among closely related yeast species (Jung *et al.* 2010; Gaillardin *et al.* 2011). The results obtained here show that this is also the case between members of the same species (Table S2). All the introns present in protein-coding genes belong to the group I intron family and encode an endonuclease, with the exception of the first intron in the *COB* gene. In the various *L. kluyveri* mt genomes studied, it is worth noting that the intron content is highly variable in the *COB* and *COX1* genes (Figure S2). The intron patterns seem to depend on the geographical origin of the strains. All the strains originating from North America contain the same two introns in the *COB* gene, whereas the number of introns ranges from two to four in the Eurasian strains (Figure S2). The first intron detected in the North American strains (between amino acids 132 and 133) occurs in 9 of the 11 Eurasian strains, whereas the second one (between amino acids 168 and 169) occurs in all the latter strains. A supplementary intron was found to exist between amino acids 143 and 144 in five of the seven European strains and two of the four Asian strains. The Asian strains also contain a specific intron located between amino acids 237 and 238, which suggests that the pattern of evolution differed. The difference in the intron content is even more pronounced in the case of the *COX1* gene. The Eurasian isolates contain four to five introns, in different combinations depending on the strains. All the Asian strains contain a first intron composed of two LAGLIDADG motifs and lack the last intron occurring in the European strains, with the exception of CBS 10367. The intron pattern of the European strains seems to be well conserved in comparison with the Asian strains, which suggests that the latter strains may have evolved independently from European ones. More surprisingly, the North American strains (62-196, CBS 6545, and CBS 6547) contain an additional intron located between amino acids 300 and 301.

All in all, these data clearly show that the intron content varied considerably between the isolates studied. This high rate of intraspecific intron variation suggests that the intron dynamics may contribute crucially to the structure of mitochondrial genomes.

### Phylogeny of the *L. kluyveri* species

**Figure 3:**
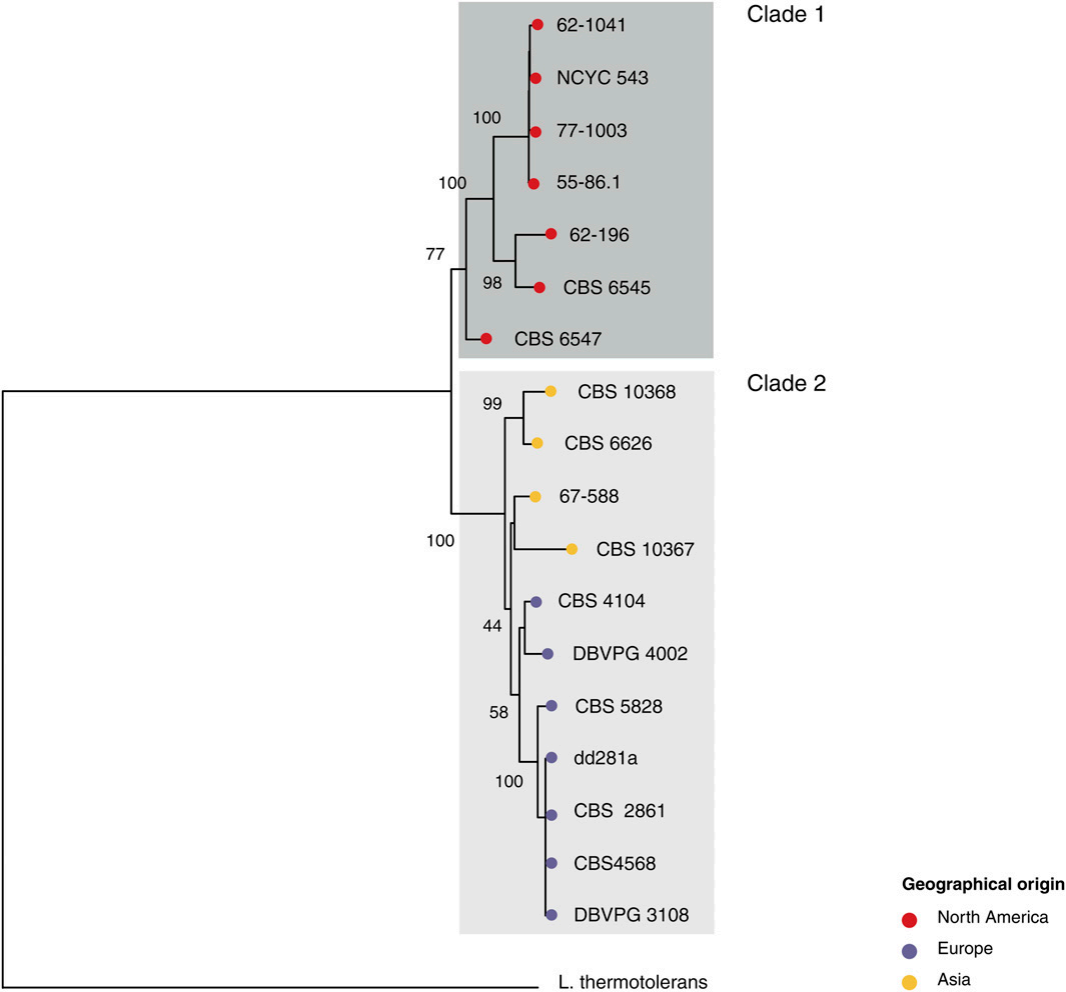
Neighbor-joining tree of 18 *L. kluyveri* strains. The tree was constructed based on the concatenation of mt genes, corresponding to 5475 polymorphic positions. Numbers are bootstrap values based on 1000 replicates.

To shed light on the intraspecific mitochondrial evolution in *L. kluyveri* and compare the effects of geographical *vs.* ecological origins on strain diversity, we investigated the phylogenetic relationships among the strains surveyed. A phylogenic tree was built based on the concatenation of the aligned *ATP6-ATP8-ATP9-COX1-COX2-COX3-COB* coding sequences. A standard neighbor-joining method was used to build a majority-rule consensus tree based on the 18 mt genomes under investigation (Figure 3). The phylogenetic tree clearly shows the existence of two tight clusters, which diverge from each other. We deduced that these two clusters consist of isolates of different geographical origins. The clades corresponding to these two clusters contain either the North American samples (clade 1) or the Eurasian samples (clade 2; Figure 3). High bootstrap values support the existence of the node between the two clusters. A similar topology was observed using two other methods (Bayesian and maximum likelihood methods) to build the tree (Figure S3). This phylogeny strongly supports the hypothesis that the evolution of the *L. kluyveri* species depended on their geographical location. By contrast, the ecological environment does not seem to have been a key factor in the evolution of *L. kluyveri*, as several strains recovered from tree exudates were found to be present in both clades.

In addition, single gene phylogenies based on the coding sequences of the two largest genes (*COB* and *COX1*) as well as the evolution of the intron pattern suggest that clade 2 could be subdivided into subclades, the first of which corresponded to the Asian strains and the second to the European ones (Figure S4). These differences probably emerged more recently than that observed between clades 1 and 2.

### Population structure provides clear-cut evidence for geographical isolation

**Figure 4:**
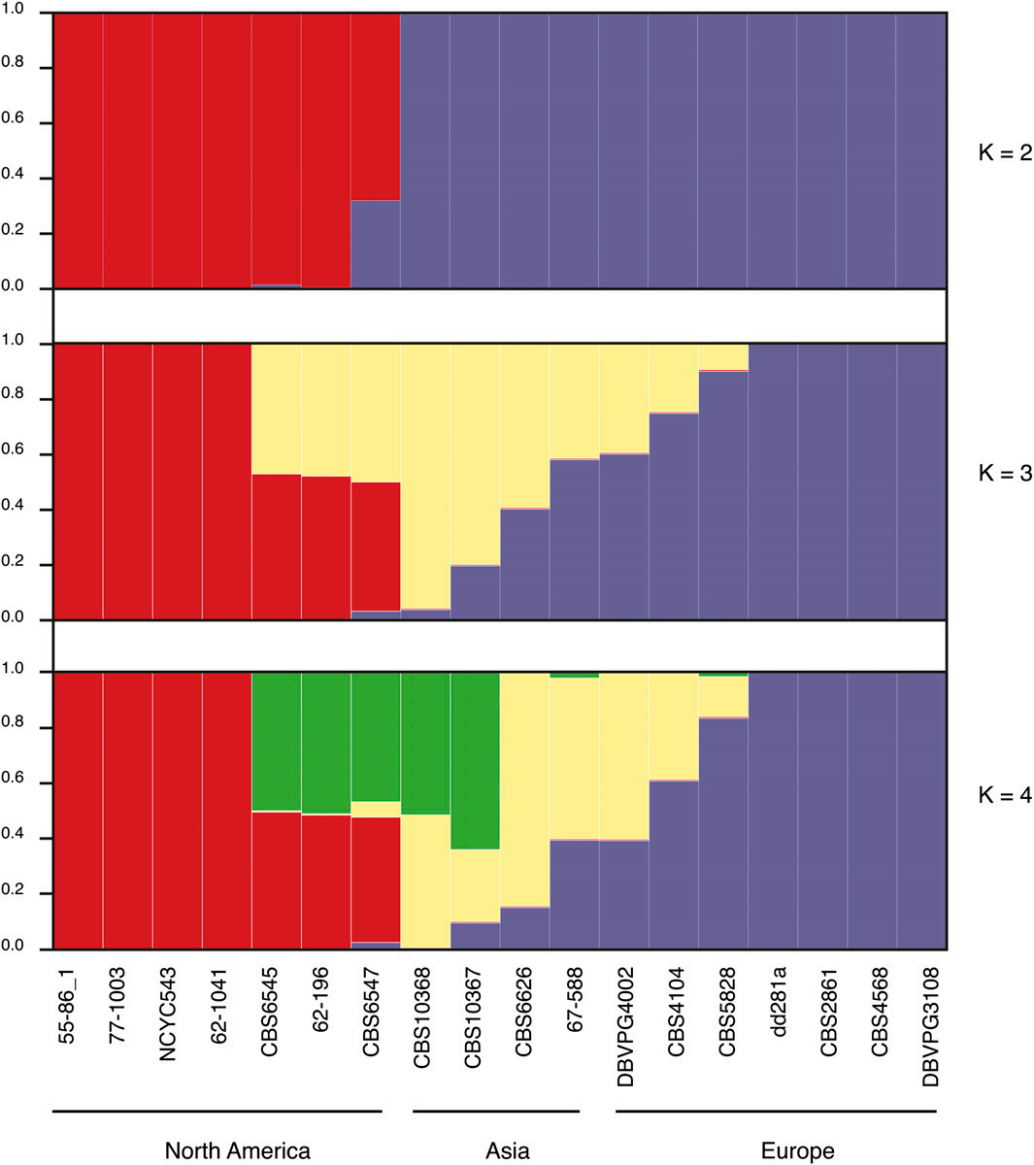
Population structure of the 18 *L. kluyveri* strains. Cluster results from a structure analysis on 180 polymorphic sites. Each strain is represented by a single vertical bar, which is partitioned into K-colored segments that represent the strain’s estimated ancestry proportion in each of the K clusters.

Phylogenetic trees do not show the true evolutionary history of strains, since they mask the effects of outcrossing among strains (Ruderfer *et al.* 2006). The population structure was therefore inferred here from the polymorphic sites across mitochondrial sequences. We used the model-based clustering algorithm implemented in the program *Structure* (Pritchard *et al.* 2000). This analysis largely confirmed the results of the phylogenetic analysis (Figure 4). The results obtained with this program were consistent with the existence of two main populations forming major geographical subgroups. The North American and Eurasian strains belong to two distinct well-defined populations (Figure 4). Some SNPs represent private polymorphism within each population, resulting in a clear-cut separation between these two populations. The CBS 6547 strain is the only one that can be said to be a mixture of two populations when the K parameter (*i.e.* the number of predicted populations) was equal to two.

However, the increase in the K parameter shows that our collection contained two clean populations. One of them is composed of four North American strains (55-86.1, 77-1003, NCYC 543, and 62-1041), whereas the second one consists of four European strains (dd281a, CBS 2861, CBS 4568, and DBVPG 3108; Figure 4). The existence of these two populations is consistent with the phylogenetic distribution previously observed, as bootstrap values of 100 were associated with both ancestral nodes. By contrast, the other 10 strains are part of mixed-populations, as they seem to be mostly composed of a combination of 2 populations. The genomes of the other three North American strains (CBS 6545, 62-196, and CBS 6547) as well as those of all the Asian strains are mosaics showing a mixed architecture. The present structural analysis clearly shows that the Asian isolates studied here are not members of a clear population. These results show that the *L. kluyveri* species is structured and composed of two clean lineages, corresponding to the North American and European populations (Figure 4).

### Whole mitochondrial genome comparisons

As suggested previously, the reason why the protein coding genes are well conserved is that purifying selection has eliminated many mutations. To investigate the mitochondrial genome-wide pattern of mitochondrial polymorphism, we compared the reference genome (NCYC 543) with four other mt genomes. Three of them belong to clade 1 (55-86.1, 77-1003, and CBS 6547) and one (CBS 5828) to clade 2 (Figure 3). Assembly of the reads yielded a single scaffold for each mt genome. As was to be expected, it turned out that exactly the same set of 35 genes is encoded by these mitochondrial genomes and that the synteny is perfectly conserved. The absence of rearrangements of the mtDNA in the *L. kluyveri* species is not surprising, in a view of the fact that practically no genomic rearrangements exist between *L. kluyveri* and *L. thermotolerans*.

However, differences in size were observed between these mt sequences (Table S2): they range from 50,137 bp in the case of CBS 6547 to 53,726 bp in that of CBS 5828. This difference can be partly explained by the difference in the intron contents mentioned previously. The North American sequences were found to have the same intron content, whereas CBS 5828 harbors three additional introns. In addition, a decrease in the size of the intergenic region was observed between the distal strains, from 41% in the case of 77−103 to 28% in that of CBS 5828 (Table S2). This difference is mainly due to the variability of the region located between the *ATP9* and *COX2* genes, which decreases in size from almost 3 kb in the case of 77−1003 to 700 bp in that of CBS 5828.

We also analyzed the genome-wide pattern of polymorphism (Table 1). The great diversity observed in the intergenic regions contrasts with the existence of highly conserved coding regions. In the coding region, the SNP density ranges from 0 to 13.96 SNPs/kb in 55−86.1 and CBS 5828, respectively. By contrast, the SNP density is much greater in the intergenic regions, where it ranges from 0.31 to 82.7 SNPs/kb in the same genomes. We also detected a total number of 1196 insertion and deletion events (indels). Single-base indels are the most common size class, and about half of them are less than five bases long in size (Figure S5). A few large deletions (up to 1695 bases in size) were detected in the CBS 6547 and CBS 5828 genomes. These strains are genetically more distantly related to the NCYC 543 reference strain than the others. Interestingly, a very large number of indels were detected in the intergenic regions. Among the 1196 indels observed, 1123 (93.9%) occur in intergenic regions, which account for only 57% of the genome on average, and only five occur in coding regions. These data suggest once again that most of the indels present in protein-coding genes were removed by selection. In addition, the density of the indels (0.19 indels/kb on average) is much lower than that of the SNPs (5.19 SNPs/kb on average) in the coding genes. This paucity of indels probably reflects the fact that most of the indels have resulted in frameshifts, and thus to a nonfunctional protein. By contrast, intergenic regions, which are mostly selectively neutral regions, show a very high density of SNPs (37.2 SNPs/kb on average) as well as indels (9.8 indels/kb on average). All in all, these data strongly suggest that most of the mutational events affecting the protein sequence have been purged by selection.

**Table 1:** Polymorphic patterns in mitochondrial genomes of 4 *L. kluyveri* isolates

	Pairwise Comparison With NCYC 543 as Reference							
	Coding Regions				Intergenic Regions			
Strains	SNPs	SNPs/kb	Indels	Indels/kb	SNPs	SNPs/kb	Indels	Indels/kb
55-86.1	0	0	0	0	9	0.31	9	0.25
77-1003	1	0.15	1	0.15	63	2.20	122	4.27
CBS 6547	44	6.68	1	0.15	1818	63.60	431	15.08
CBS 5828	92	13.96	3	0.46	2364	82.70	561	19.62

SNP, single-nucleotide polymorphism.

## Discussion

The availability of mt genomes from single isolates of various hemiascomycetous yeast species has improved our understanding of mtDNA evolution at different levels (Eldarov *et al.* 2011; Jung *et al.* 2010; Kosa *et al.* 2006; Pramateftaki *et al.* 2006; Prochazka *et al.* 2010). Comparative genomic studies on these genomes have shown the existence of many differences in their structure, organization, and topology (Solieri 2010). However, no comparisons have been carried out so far on single yeast species. Some whole-mtDNA genome studies recently shed interesting light on selection and evolution in humans (Batini *et al.* 2011; Elson *et al.* 2004; Gunnarsdóttir *et al.* 2011, Schönberg *et al.* 2011). To investigate the genetic diversity and the patterns of mitochondrial genome evolution occurring in a single yeast species, we sequenced the complete mtDNA of 18 *L. kluyveri* isolates. In addition, the data we generated will help us to have a better understanding of phenotypic variation linked mtDNA diversity.

One aim of this study was to assess the evolutionary history of the *L. kluyveri* species. Mitochondrial coding sequences of the 18 isolates were used to investigate the phylogeny by various methods. All the phylogenetic analyses clearly showed that the genomes sequenced comprised two tight clusters, corresponding to the North American and Eurasian isolates (Figure 3). To investigate the evolutionary history of these strains more closely, the population structure was also determined and the data obtained yielded some useful information about the populations in question (Figure 4). They also suggested that the genetic variations observed in *L. kluyveri* have a geographical basis. Evolutionary patterns based on geographical origin have previously been described, especially in the *S. paradoxus* species (Liti *et al.* 2009). Studies on the variability of this species clearly showed the existence of four populations originating from Europe, the Far East, America, and Hawaii (Liti *et al.* 2009). The evolution of *S. cerevisiae* strains was found on the contrary to be highly correlated with the ecological niches from which they originated rather than with their geographical origins (Schacherer *et al.* 2009). A clear-cut population structure was observed in the main ecological subgroups. These populations result from separate domestication events, but *S. cerevisiae* as a whole is not a domesticated species (Liti *et al.* 2009).

All these data give an overall picture of the intraspecific variability of the yeast mt genome. In terms of their size and intron content, the yeast mt genomes of this species are highly variable (Table S2). The size ranged from 50.1 to 53.7 kb in CBS 6547 and CBS 5828, respectively. These differences may be attributable to either the intron content or the length of the intergenic regions. In fact, there exists a balance between the variations in these two regions. The size of intergenic regions varies between 15 to 21.2 kb, whereas the intron content varies both in number (between 6 and 9) and size (between 8.7 kb and 12.8 kb; Table S2). The large genome size of the CBS 5828 isolate can be explained, for example, by the presence of nine introns, amounting to 12.8 kb. All the coding introns identified in the various yeast genomes studied here belong to the group I introns and encode endonucleases. It was established long time ago that group I introns are mobile elements, which can be gained or lost (Delahodde *et al.* 1989; Dujon *et al.* 1989; Wenzlau *et al.* 1989). The patterns of intron content observed in the wild isolates are interesting because they indicate that the mt genomes show a high level of intraspecific plasticity (Figure S2). These patterns are correlated with the phylogeny of the strains and are therefore similar between strains originating from similar geographical locations. In addition to the difference in size and intron content, the gene content and order are conserved across the strains, as suggested by previous interspecific genome comparisons.

Whole-mtDNA genome studies also provide a good means of addressing selection issues. These issues have been extensively studied in humans (Batini *et al.* 2011; Gunnarsdóttir *et al.* 2011; Schönberg *et al.* 2011). To investigate the patterns of polymorphism involved in mitochondrial genome evolution among *L. kluyveri* isolates, we quantified the selective constraints to which each of the mitochondrial genes was exposed by estimating ω. These ratios were found to be very low and consistent among the genes studied, with the noteworthy exception of the *VAR1* gene, which gave a very high ratio. The low dN/dS ratios of most of the other mitochondrial genes provide evidence for purifying selection, *i.e.*, most of the amino acid substitutions which occurred were deleterious and were therefore removed. Despite this purifying selection, we found definite evidence for relaxed purifying selection for two genes. First, the *ATP6* gene is characterized by a greater than average ω value (median value, 0.21). The *ATP6* gene is one of three mitochondrial genes, the others being *ATP8* and *ATP9*, which encode ATP synthase subunits. The most interesting point here, however, is the particularly high ω value obtained in the case of the *VAR1* gene (median value: 0.35). The *VAR1* gene encodes a mitochondrial ribosomal protein of the small subunit. Interestingly, *VAR1* is not found in all the yeast mitochondrial genomes, and shows a scattered pattern of distribution among hemiascomycetous yeasts (Jung *et al.* 2009). The most parsimonious explanation for the relaxation of the purifying selection should be a reduced functional constraint in the *VAR1* gene.

Genome-wide patterns of SNPs and indels were also studied. Large disparities were noted between the distribution of the polymorphisms observed in the coding and intergenic regions (Table 1). The mean density of nucleotide polymorphism was estimated at 5.2 SNPs/kb in the coding region and 37.2 SNPs/kb in the intergenic regions. This high figure is also consistent with the effects of purifying selection. This selection was also reflected in the high indel density in the intergenic regions (9.8 indels/kb on average), whereas they amounted to only 0.19 indels/kb on average in the coding regions. These data suggest once again that most of the indels in protein-coding genes were removed by selection. In addition, it is worth noting that most of the indels were found to be single-base or very small indels (Figure S5). The fact that indels are mostly concentrated in homopolymers also suggests that sequence variations may have been caused by replication slippage events (Behura 2007, Noutsos *et al.* 2005).

The overall density of nucleotide polymorphism of the mt genomes studied here is very high, amounting 28.5 SNPs/kb on average, which is 10-fold greater than that previously observed in the nuclear genome of *S. cerevisiae* (2.8 SNPs/kb on average) (Schacherer *et al.* 2009). This finding seems to confirm that the mtDNA shows a higher mutation rate than the nuclear genome (Clark-Walker 1991; Lynch and Blanchard 1998; Lynch *et al.* 2006; Lynch 2007). In *S. cerevisiae*, mutation-accumulation lines have been used to estimate the rate of nucleotide substitution in nuclear and mt genomes (Lynch *et al.* 2008). Nucleotide substitution rates have been estimated to be 37-fold greater in the mitochondria than in the nuclei. There are several explanations for this high mutation rate. First, mtDNA undergoes more replication and may accumulate more mutations than nuclear DNA explaining the hypermutability state of the organelle. In *S. cerevisiae*, the mtDNA genome is present normally at 20−100 copies per cell comprising 10–20% of the total cellular DNA (Williamson and Fennell 1979). Second, there is a low efficient of the DNA repair pathway (Song *et al.* 2005). Finally, the intracellular environment is more mutagenic (Lynch 2007).

The intraspecific diversity of the yeast mt genome was described here in detail for the first time. As mentioned previously, the content and order of the genes have been completely conserved. By contrast, the size, intron content and the intergenic regions were found to vary from one isolates to another. The pattern of variation observed among the mitochondrial genes, with the exception of the *ATP6* and *VAR1* genes, is consistent with purifying selection. It would now be interesting to determine the rates and patterns of mtDNA in close relatives of this species such as *Lachancea cidri*, *Lachancea fermentati*, *Lachancea meyersii*, and *Lachancea waltii* (Naumova *et al.* 2007). Such a study could address the issues of selection and compare the patterns of mt sequence variation at two hierarchical levels: within species and among closely related species.

## Supplementary Material

Supporting Information
