# Supplementary material for: Mitochondrial Genome Evolution in a Single Protoploid Yeast Species
Source: G3 (Bethesda). 2012 Sep 1;2(9):1103–11. doi: 10.1534/g3.112.003152 (PMC3429925; doi:10.1534/g3.112.003152)
Supplement: Supporting Information [file supp_2_9_1103__index.html]

Supporting Information 

# Mitochondrial Genome Evolution in a Single Protoploid Yeast Species

## Supporting Information for Jung *et al.*, 2012

**Files in this Data Supplement:**

- Supporting Information - Figures S1-S5 and Tables S1-S3 (PDF, 239 KB)
- Figure S1 - Box-plot comparisons of dN and dS substitution rates estimated in the various mt genes, and based on pairwise alignments between the NCYC 543 strains and the other strains studied (PDF, 72 KB)
- Figure S2 - Intron variability in *COX1* and *COB genes*. All the coding introns found in these two genes belong to the LAGLIDADG superfamily of group I introns and are presented in the form of orange circles (PDF, 113 KB)
- Figure S3 - Phylogeny of *L. kluyveri* strains obtained using Bayesian (a) and Maximum-Likelihood (b) methods, based on the concatenation of mt genes, amounting a total number of 5,475 positions (PDF, 72 KB)
- Figure S4 - Phylogeny of *L. kluyveri* strains using the Neighbor-Joining method, based on the nucleotide sequences of *COX1* (a) and *COB* (b) genes. Numbers are bootstrap values obtained on 1,000 replicates (PDF, 74 KB)
- Figure S5 - Size of the indels in the mt genome studied (PDF, 88 KB)
- Table S1 - Description of *L. kluyveri* strains studied (PDF, 68 KB)
- Table S2 - General features of the mitochondrial genomes (PDF, 55 KB)
- Table S3 - Pairwise non-synonymous/synonymous differences relative to NCYC 543 (PDF, 54 KB)
