# Supplementary material for: Mitochondrial Genome Evolution in a Single Protoploid Yeast Species
Source: G3 (Bethesda). 2012 Sep 1;2(9):1103–11. doi: 10.1534/g3.112.003152 (PMC3429925; doi:10.1534/g3.112.003152)
Supplement: Supporting Information [file supp_2.9.1103_003152SI.pdf]

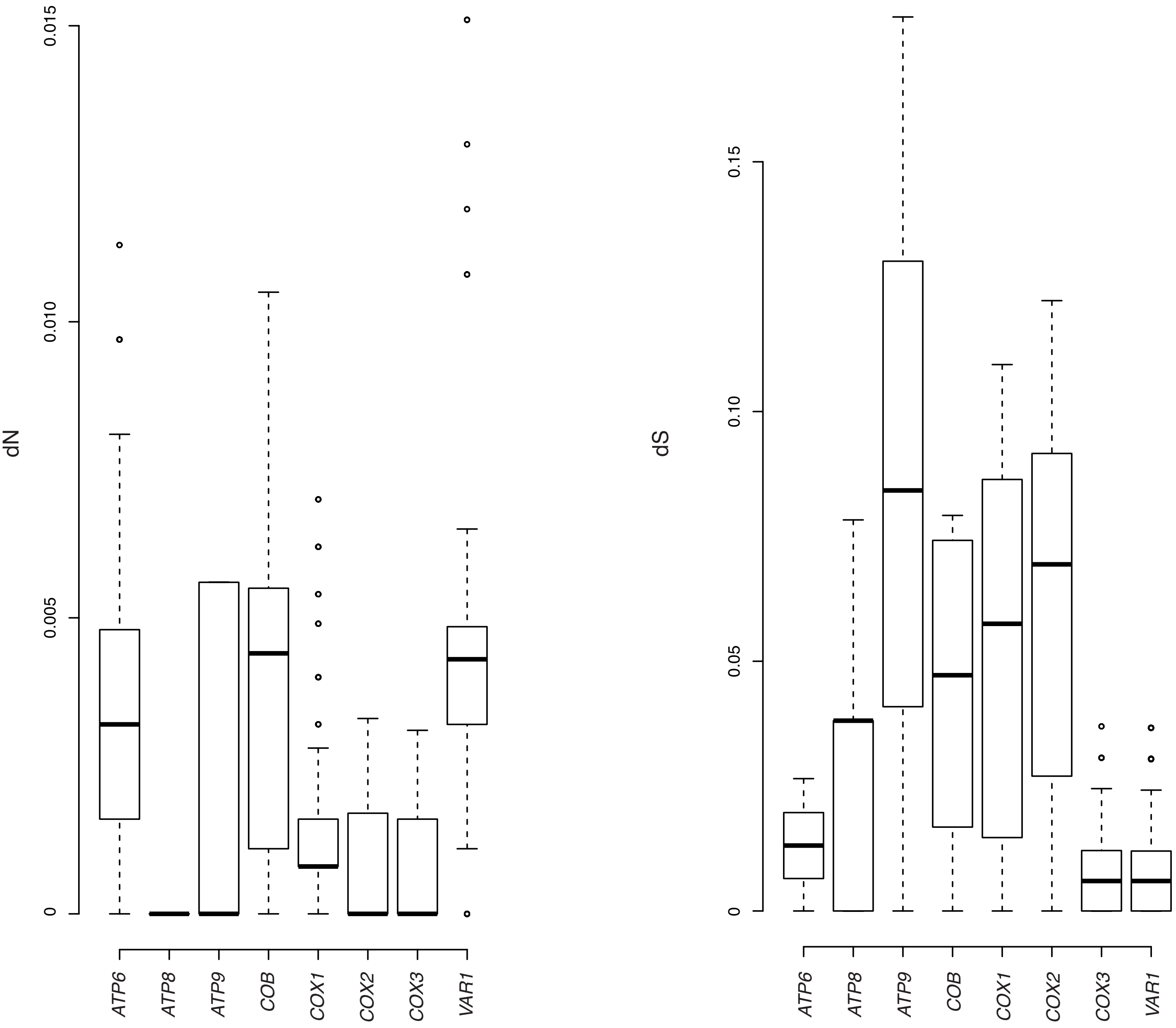

Figure S1 Box-plot comparisons of dN and dS substitution rates estimated in the various mt genes, and based on pairwise alignments between the NCYC 543 strains and the other strains studied.

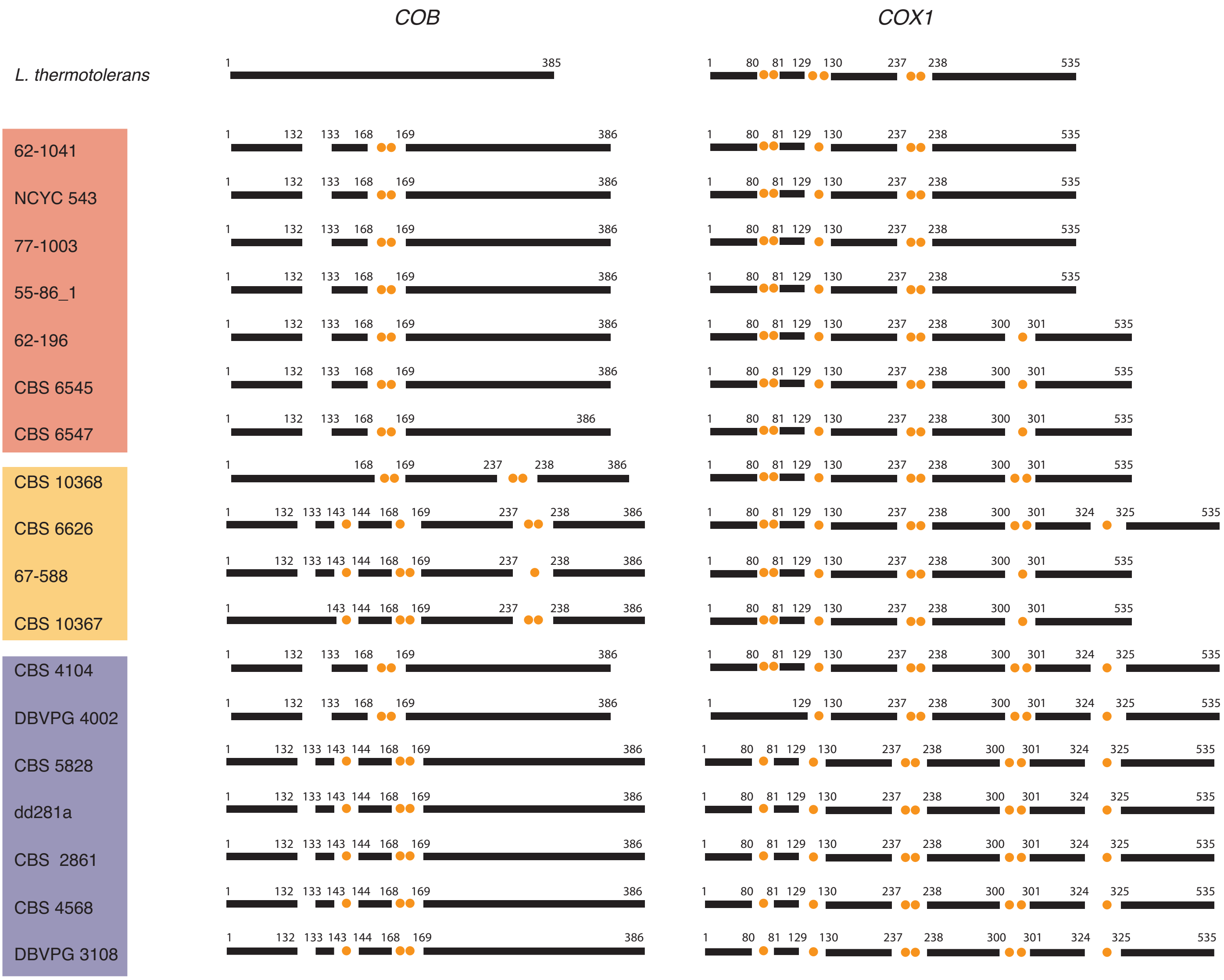

Figure S2 Intron variability in *COX1* and *COB* genes. All the coding introns found in these two genes belong to the LAGLIDADG superfamily of group I introns and are presented in the form of orange circles. Numbers of circles depend on the number of LAGLIDADG motifs. Numbers are the coordinates of the corresponding proteins.

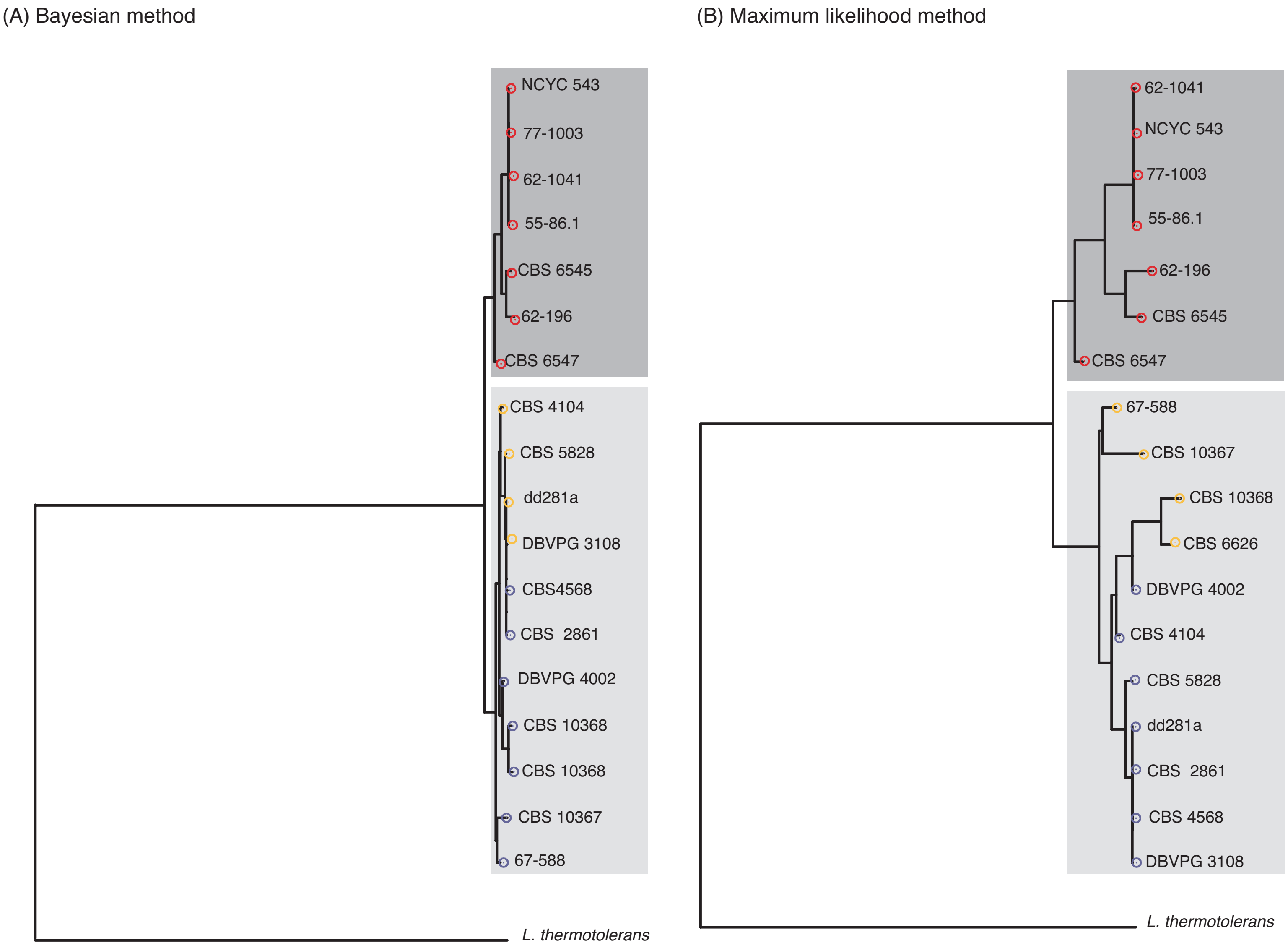

Figure S3 Phylogeny of *L. kluyveri* strains obtained using Bayesian (a) and Maximum-Likelihood (b) methods, based on the concatenation of mt genes, amounting a total number of 5,475 positions.

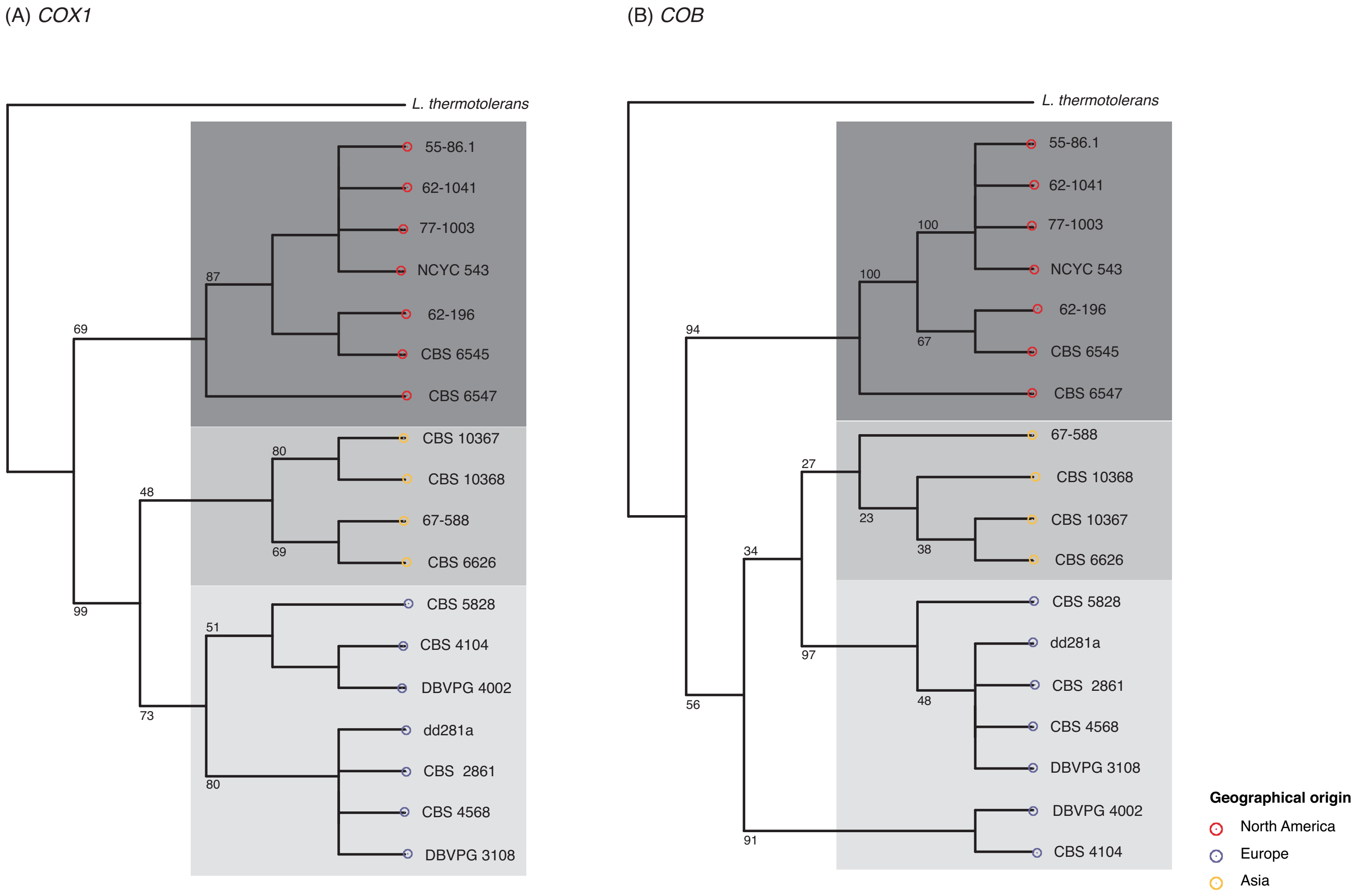

Figure S4 Phylogeny of *L. kluyveri* strains using the Neighbor-Joining method, based on the nucleotide sequences of *COX1* (a) and *COB* (b) genes. Numbers are bootstrap values obtained on 1,000 replicates.

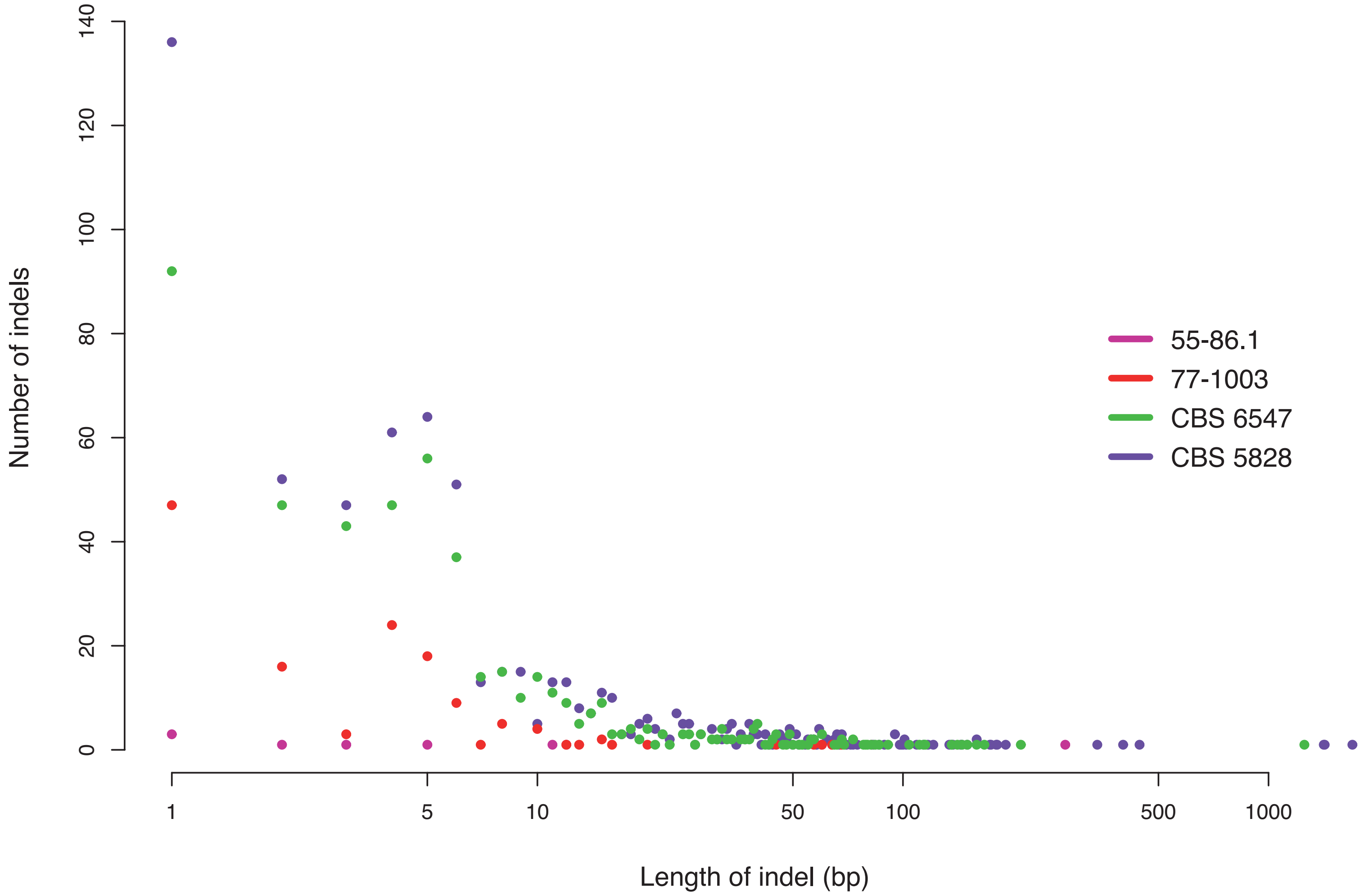

Figure S5 Size of the indels in the mt genome studied.

Table S1 Description of *L. kluyveri* strains studied

Strains	Sources	Location
55-86.1	Salix exudate	USA, California
62-1041	Willow exudate	USA, California, Davis
62-196	Taraxacum officinale	Canada, Saskatoon
67-588	Exudate of *Ulmus japonica*	Japan, Yamabe
77-1003	Unknown	USA, California
CBS 2861	Soil	Sweden
CBS 4104	Soil	Netherlands, Wageningen
CBS 4568	Soil	Sweden
CBS 5828	Soil	Denmark
CBS 6545	Salix exudate	USA, California
CBS 6547	*Drosophila pseudobscura*	USA, California, Gualala Creek
CBS 6626	Exudate of tree	Japan, Mt. Takamatsu
CBS 10367	*Quercus mongolica* exudate	Russia, Kedrova pad nature reserve
CBS 10368	*Quercus mongolica* exudate	Russia, Sikhote-Alinsky nature reserve
DBVPG 3108	Soil	Netherlands
DBVPG 4002	Cavern	Italy
dd.281a	Forest	Germany, Heidelberg
NCYC 543	*Drosophila pinicola*	USA, California

Table S2 General features of the mitochondrial genomes

Species	Strains	Size (bp)	GC %	CDS (%)	intron (%)	tRNA (%)	rRNA (%)	Intergenic region (%)	Intron number	Total intron size (bp)
L. thermotolerans	CBS 6340	23,584	24.8	29.9	18.3	7.6	21.9	22.3	3	4,320
L. kluyveri	NCYC 543	51,525	15.1	29	17	3.5	9.5	41	6	8,777
	55-86.1	51,465	15.1	29.1	17.1	3.5	9.5	40.8	6	8,787
	77-1003	51,679	15.2	29	17.1	3.5	9.4	41	6	8,854
	CBS 5828	53,726	17.3	35.6	23.8	3.3	9.3	28	9	12,795
	CBS 6547	50,137	16.8	30.4	18.1	3.6	9.7	38.2	7	9,058

Table S3 Pairwise non-synonymous/synonymous differences relative to NCYC 543

Genes	Length (bp)	GC %	Non-synonymous/synonymous differences relative to NCYC 543																
			62-1041	77-1003	55-86_1	62-196	CBS 6545	CBS 6547	CBS 10368	CBS 6626	67-588	CBS 10367	CBS 4104	DBVPG 4002	CBS 5828	dd281 a	CBS 2861	CBS 4568	DBVPG 3108
ATP6	777	23	0/0	0/0	0/0	4/4	2/3	1/2	6/3	3/3	1/1	1/1	1/3	3/4	2/3	2/3	2/3	2/3	2/3
ATP8	144	20	0/0	0/0	0/0	0/1	0/1	0/1	0/1	0/1	0/1	0/1	0/1	0/1	0/1	0/1	0/1	0/1	0/1
ATP9	228	32.9	0/0	1/0	0/0	0/4	0/6	0/5	1/8	1/8	0/5	0/5	0/6	1/8	0/6	0/6	0/6	0/6	0/6
COB	1,155	27.5	0/0	0/0	0/0	1/4	1/6	2/11	1/17	3/17	1/18	8/19	1/16	1/16	6/18	5/19	5/19	5/19	5/19
COX1	1,602	29.5	0/0	0/0	0/0	2/11	2/12	2/10	4/31	2/29	2/28	5/33	1/28	1/26	1/27	1/28	1/28	1/28	1/28
COX2	753	26.2	0/0	0/0	0/0	2/4	1/2	1/4	1/14	1/13	1/17	1/15	1/15	1/17	1/11	1/13	1/13	1/13	1/13
COX3	807	27.2	0/0	0/0	0/0	1/3	0/3	0/1	0/2	0/2	0/3	1/1	0/2	0/2	0/2	0/2	0/2	0/2	0/2
VAR1	1,125	10.7	0/0	0/0	0/0	4/3	10/6	2/0	-	2/3	3/1	3/3	3/2	3/2	5/2	3/3	3/3	3/3	3/3
