# Supplementary material for: Mitochondrial Genome Evolution in a Single Protoploid Yeast Species
Source: G3 (Bethesda). 2012 Sep 1;2(9):1103–11. doi: 10.1534/g3.112.003152 (PMC3429925; doi:10.1534/g3.112.003152)
Supplement: Supporting Information [file supp_2.9.1103_FigureS1.pdf]

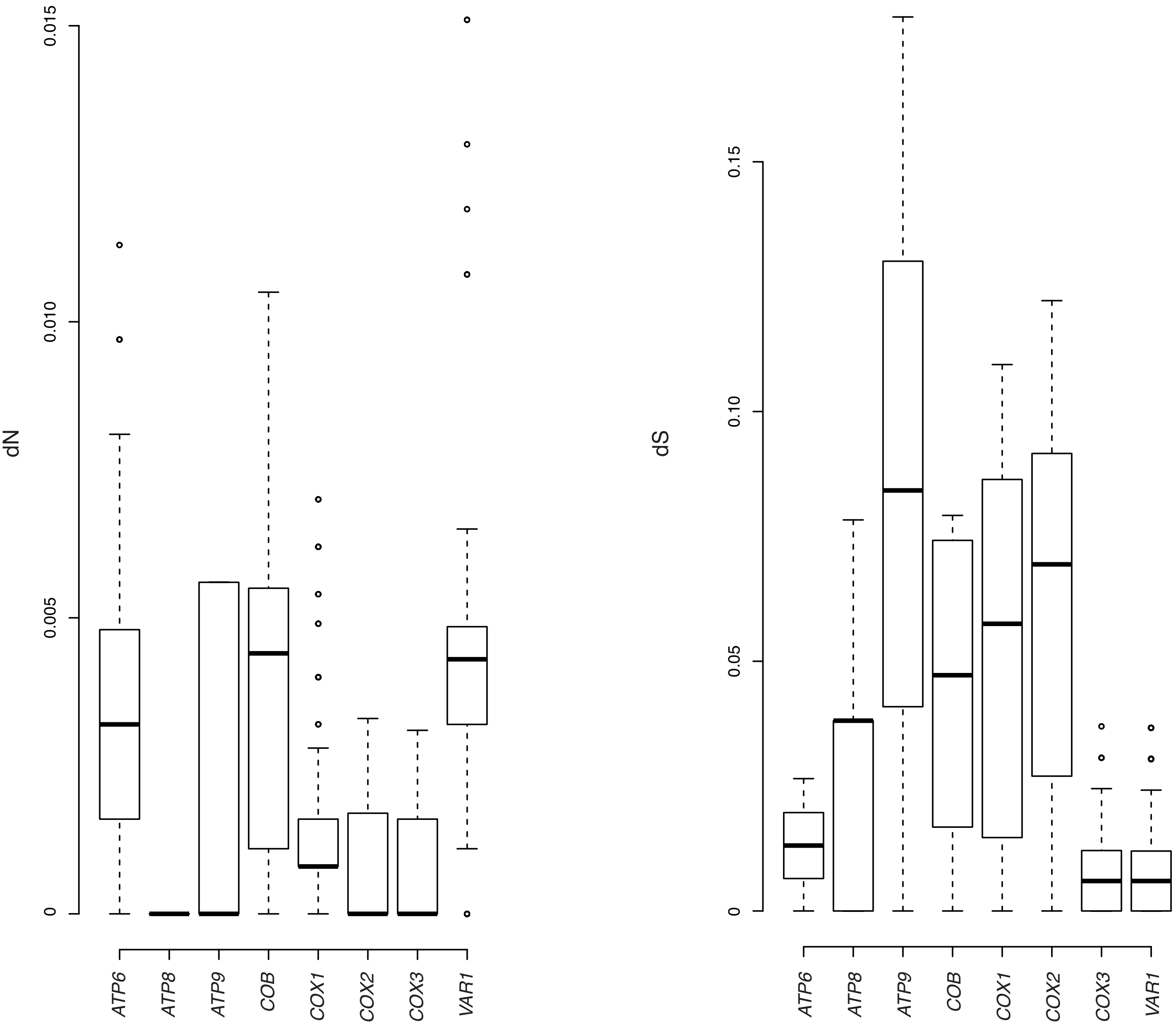

Figure S1 Box-plot comparisons of dN and dS substitution rates estimated in the various mt genes, and based on pairwise alignments between the NCYC 543 strains and the other strains studied.
