# Supplementary material for: Mitochondrial Genome Evolution in a Single Protoploid Yeast Species
Source: G3 (Bethesda). 2012 Sep 1;2(9):1103–11. doi: 10.1534/g3.112.003152 (PMC3429925; doi:10.1534/g3.112.003152)
Supplement: Supporting Information [file supp_2.9.1103_FigureS2.pdf]

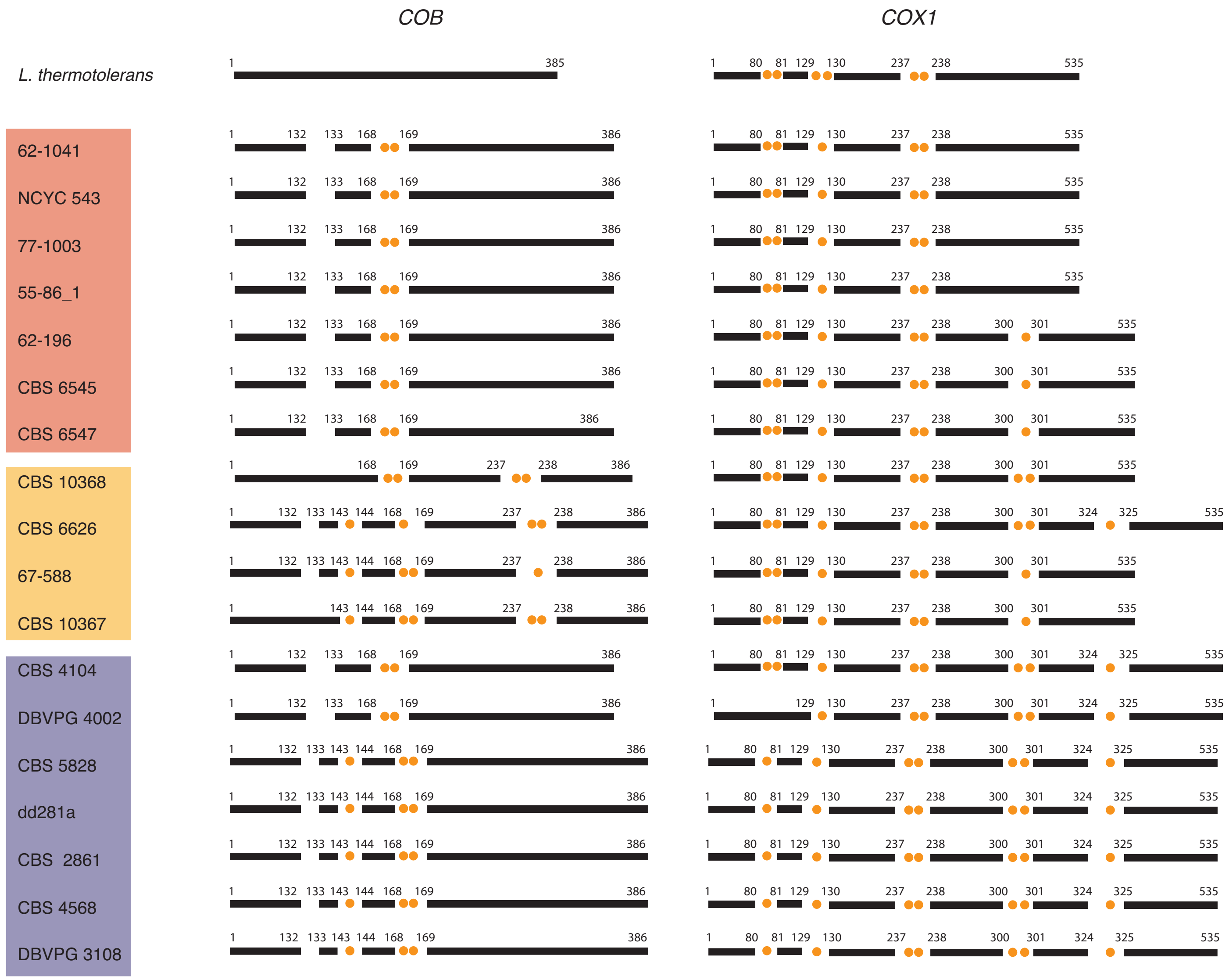


**Figure S2** Intron variability in *COX1* and *COB* genes. All the coding introns found in these two genes belong to the LAGLIDADG superfamily of group I introns and are presented in the form of orange circles. Numbers of circles depend on the number of LAGLIDADG motifs. Numbers are the coordinates of the corresponding proteins.
