# Supplementary material for: Mitochondrial Genome Evolution in a Single Protoploid Yeast Species
Source: G3 (Bethesda). 2012 Sep 1;2(9):1103–11. doi: 10.1534/g3.112.003152 (PMC3429925; doi:10.1534/g3.112.003152)
Supplement: Supporting Information [file supp_2.9.1103_FigureS3.pdf]

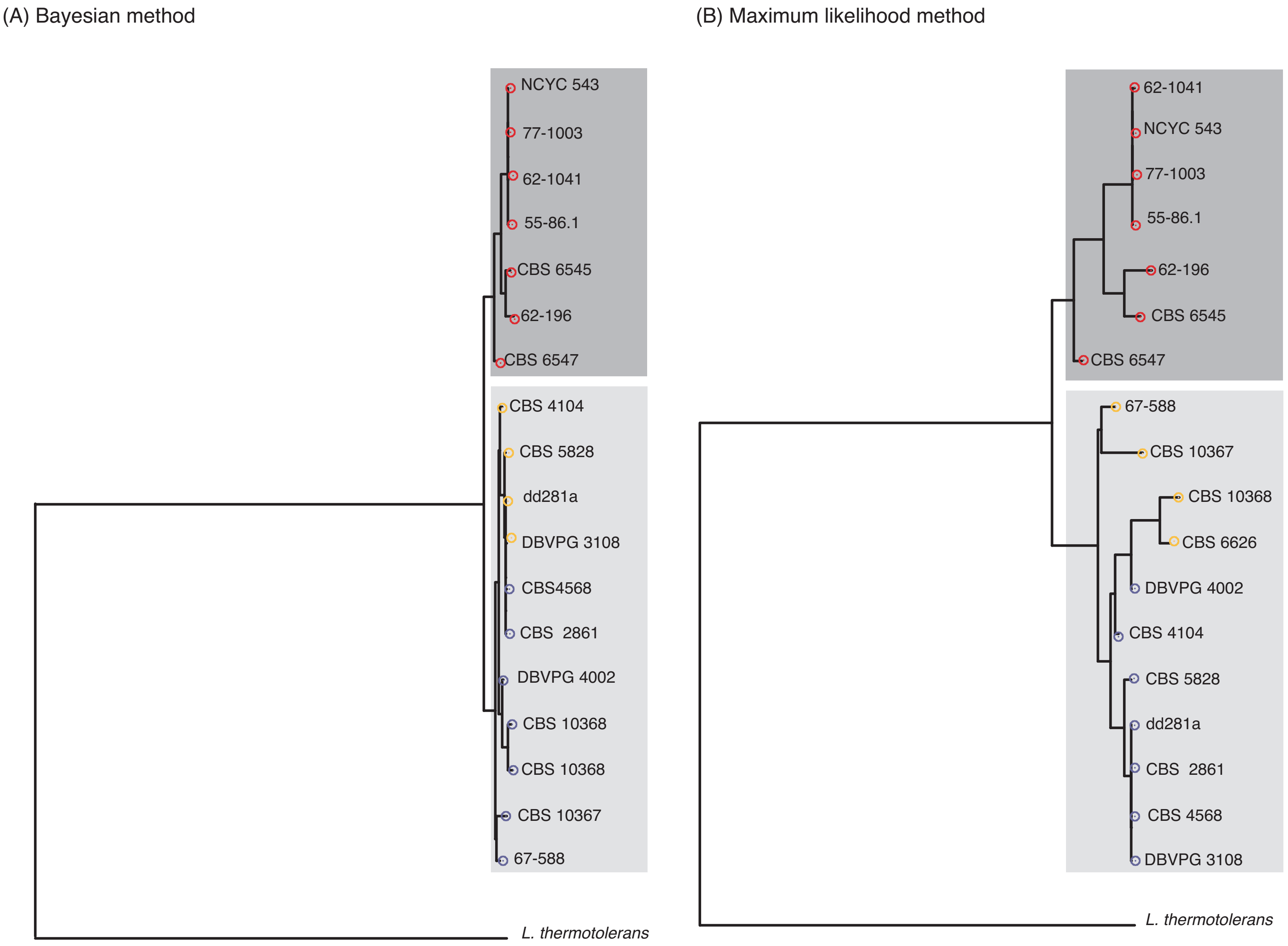


**Figure S3** Phylogeny of *L. kluyveri* strains obtained using Bayesian (a) and Maximum-Likelihood (b) methods, based on the concatenation of mt genes, amounting a total number of 5,475 positions.
