# Supplementary material for: Mitochondrial Genome Evolution in a Single Protoploid Yeast Species
Source: G3 (Bethesda). 2012 Sep 1;2(9):1103–11. doi: 10.1534/g3.112.003152 (PMC3429925; doi:10.1534/g3.112.003152)
Supplement: Supporting Information [file supp_2.9.1103_FigureS4.pdf]

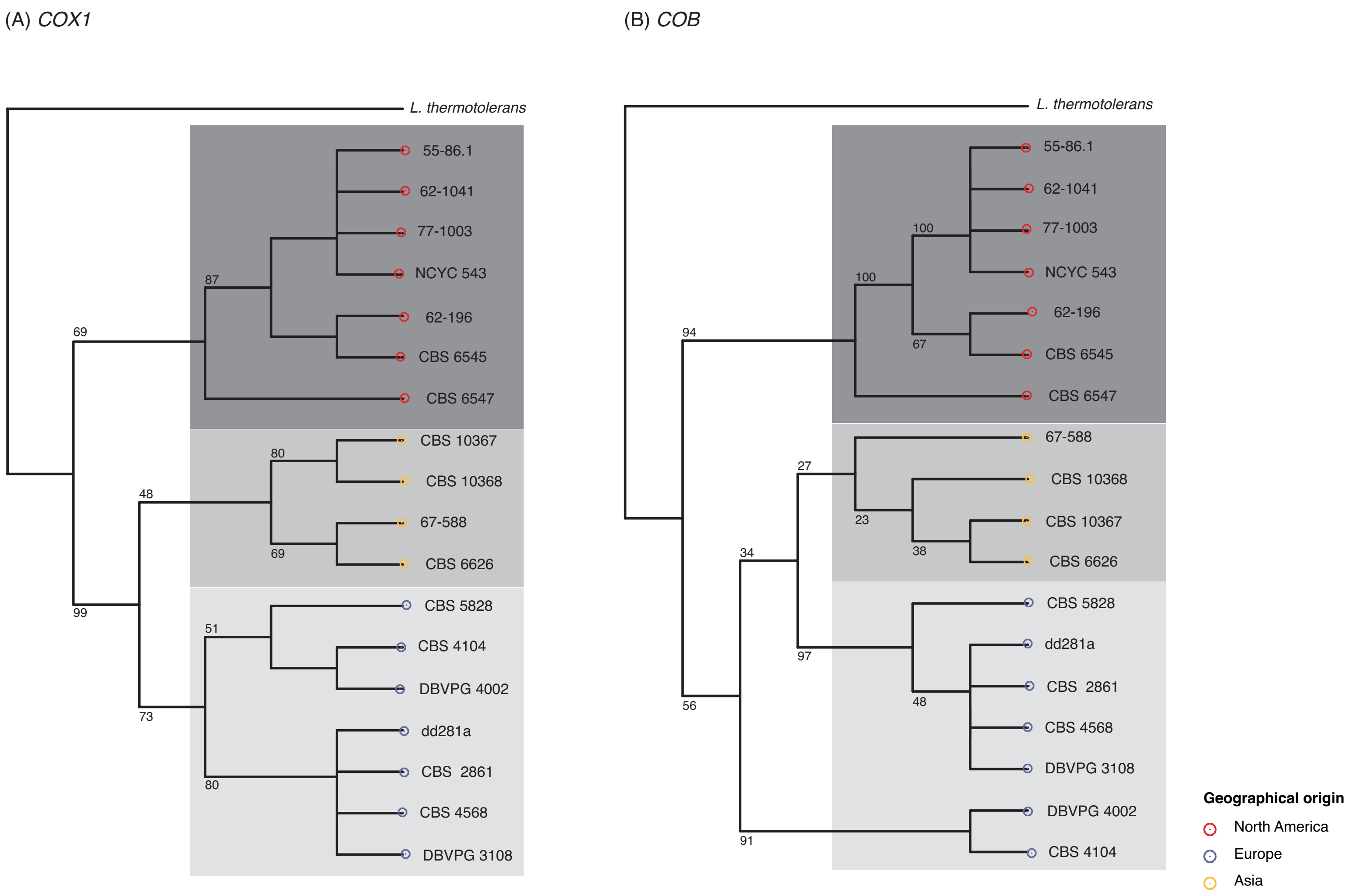

Figure S4 Phylogeny of *L. kluyveri* strains using the Neighbor-Joining method, based on the nucleotide sequences of *COX1* (a) and *COB* (b) genes. Numbers are bootstrap values obtained on 1,000 replicates.
