# Supplementary figures and images for: Mitochondrial Genome Evolution in a Single Protoploid Yeast Species
Source: G3 (Bethesda). 2012 Sep 1;2(9):1103–11. doi: 10.1534/g3.112.003152 (PMC3429925; doi:10.1534/g3.112.003152)

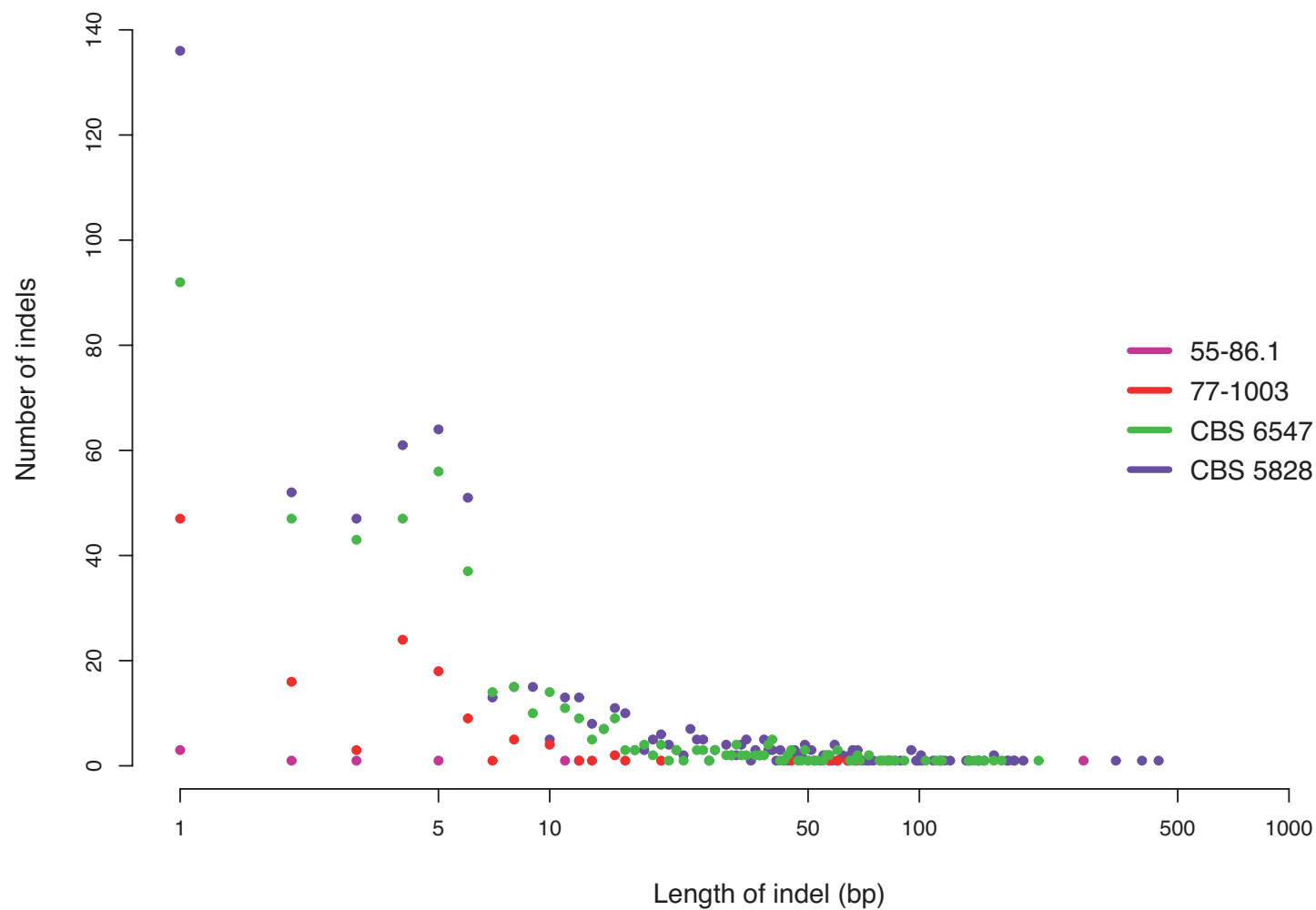

**Figure S5** Size of the indels in the mt genome studied.

Supplement: Supporting Information [file supp_2.9.1103_FigureS5.pdf]
