# Supplementary material for: Mitochondrial Genome Evolution in a Single Protoploid Yeast Species
Source: G3 (Bethesda). 2012 Sep 1;2(9):1103–11. doi: 10.1534/g3.112.003152 (PMC3429925; doi:10.1534/g3.112.003152)
Supplement: Supporting Information [file supp_2.9.1103_TableS1.pdf]

Table S1 Description of *L. kluyveri* strains studied

Strains	Sources	Location
55-86.1	Salix exudate	USA, California
62-1041	Willow exudate	USA, California, Davis
62-196	Taraxacum officinale	Canada, Saskatoon
67-588	Exudate of *Ulmus japonica*	Japan, Yamabe
77-1003	Unknown	USA, California
CBS 2861	Soil	Sweden
CBS 4104	Soil	Netherlands, Wageningen
CBS 4568	Soil	Sweden
CBS 5828	Soil	Denmark
CBS 6545	Salix exudate	USA, California
CBS 6547	*Drosophila pseudobscura*	USA, California, Gualala Creek
CBS 6626	Exudate of tree	Japan, Mt. Takamatsu
CBS 10367	*Quercus mongolica* exudate	Russia, Kedrova pad nature reserve
CBS 10368	*Quercus mongolica* exudate	Russia, Sikhote-Alinsky nature reserve
DBVPG 3108	Soil	Netherlands
DBVPG 4002	Cavern	Italy
dd.281a	Forest	Germany, Heidelberg
NCYC 543	*Drosophila pinicola*	USA, California
