# Supplementary material for: Mitochondrial Genome Evolution in a Single Protoploid Yeast Species
Source: G3 (Bethesda). 2012 Sep 1;2(9):1103–11. doi: 10.1534/g3.112.003152 (PMC3429925; doi:10.1534/g3.112.003152)
Supplement: Supporting Information [file supp_2.9.1103_TableS2.pdf]

Table S2 General features of the mitochondrial genomes

Species	Strains	Size (bp)	GC %	CDS (%)	intron (%)	tRNA (%)	rRNA (%)	Intergenic region (%)	Intron number	Total intron size (bp)
L. thermotolerans	CBS 6340	23,584	24.8	29.9	18.3	7.6	21.9	22.3	3	4,320
L. kluyveri	NCYC 543	51,525	15.1	29	17	3.5	9.5	41	6	8,777
	55-86.1	51,465	15.1	29.1	17.1	3.5	9.5	40.8	6	8,787
	77-1003	51,679	15.2	29	17.1	3.5	9.4	41	6	8,854
	CBS 5828	53,726	17.3	35.6	23.8	3.3	9.3	28	9	12,795
	CBS 6547	50,137	16.8	30.4	18.1	3.6	9.7	38.2	7	9,058
