# Supplementary material for: Mitochondrial Genome Evolution in a Single Protoploid Yeast Species
Source: G3 (Bethesda). 2012 Sep 1;2(9):1103–11. doi: 10.1534/g3.112.003152 (PMC3429925; doi:10.1534/g3.112.003152)
Supplement: Supporting Information [file supp_2.9.1103_TableS3.pdf]

Table S3 Pairwise non-synonymous/synonymous differences relative to NCYC 543

Genes	Length (bp)	GC %	Non-synonymous/synonymous differences relative to NCYC 543																
			62-1041	77-1003	55-86_1	62-196	CBS 6545	CBS 6547	CBS 10368	CBS 6626	67-588	CBS 10367	CBS 4104	DBVPG 4002	CBS 5828	dd281a	CBS 2861	CBS 4568	DBVPG 3108
ATP6	777	23	0/0	0/0	0/0	4/4	2/3	1/2	6/3	3/3	1/1	1/1	1/3	3/4	2/3	2/3	2/3	2/3	2/3
ATP8	144	20	0/0	0/0	0/0	0/1	0/1	0/1	0/1	0/1	0/1	0/1	0/1	0/1	0/1	0/1	0/1	0/1	0/1
ATP9	228	32.9	0/0	1/0	0/0	0/4	0/6	0/5	1/8	1/8	0/5	0/5	0/6	1/8	0/6	0/6	0/6	0/6	0/6
COB	1,155	27.5	0/0	0/0	0/0	1/4	1/6	2/11	1/17	3/17	1/18	8/19	1/16	1/16	6/18	5/19	5/19	5/19	5/19
COX1	1,602	29.5	0/0	0/0	0/0	2/11	2/12	2/10	4/31	2/29	2/28	5/33	1/28	1/26	1/27	1/28	1/28	1/28	1/28
COX2	753	26.2	0/0	0/0	0/0	2/4	1/2	1/4	1/14	1/13	1/17	1/15	1/15	1/17	1/11	1/13	1/13	1/13	1/13
COX3	807	27.2	0/0	0/0	0/0	1/3	0/3	0/1	0/2	0/2	0/3	1/1	0/2	0/2	0/2	0/2	0/2	0/2	0/2
VAR1	1,125	10.7	0/0	0/0	0/0	4/3	10/6	2/0	-	2/3	3/1	3/3	3/2	3/2	5/2	3/3	3/3	3/3	3/3
